# Genetic determination of the effect of post-translational modification on the innate immune response to the 19 kDa lipoprotein of *Mycobacterium tuberculosis*

**DOI:** 10.1186/1471-2180-9-93

**Published:** 2009-05-14

**Authors:** Katalin A Wilkinson, Sandra M Newton, Graham R Stewart, Adrian R Martineau, Janisha Patel, Susan M Sullivan, Jean-Louis Herrmann, Olivier Neyrolles, Douglas B Young, Robert J Wilkinson

**Affiliations:** 1 National Institute for Medical Research, Mill Hill, London, NW7 1AA, UK; 2 Faculty of Health and Medical Sciences, University of Surrey, Guildford, UK; 3 Brighton and Sussex Medical School, Brighton, UK; 4 Department of Molecular, Cellular and Developmental Biology, University of Michigan, Ann Arbor, Michigan, 48109, USA; 5 Service de Microbiologie, Hopital Saint Louis, 1, avenue Claude-Vellefaux, 75475 Paris Cedex 10, France; 6 Université Paul Sabatier and CNRS, Institut de Pharmacologie et de Biologie Structurale (UMR 5089) 31077 Toulouse, Cedex 4, France; 7 Division of Medicine and Centre for Molecular Microbiology and Infection, Imperial College London, London, UK; 8 Institute of Infectious Diseases and Molecular Medicine, Faculty of Health Sciences, University of Cape Town, Observatory 7925, South Africa; 9 Current address: Centre for Health Sciences, Queen Mary's School of Medicine and Dentistry, 2 Newark St, London, E1 2AT, UK

## Abstract

**Background:**

The 19 kDa lipoprotein of *Mycobacterium tuberculosis *(MTB) is an important target of the innate immune response. To investigate the effect of post-translation modification of this protein on innate recognition in the context of the whole bacillus, we derived a recombinant *M. tuberculosis *H37Rv that lacked the 19 kDa gene (Δ19) and complemented this strain by reintroduction of the 19 kDa gene into the chromosome as a single copy to produce Δ19::19. We also reintroduced the 19 kDa gene in two modified forms that lacked motifs for acylation (Δ19::19NA) and *O*-glycosylation (Δ19::19NOG).

**Results:**

Both acylation and *O*-glycosylation were necessary for the protein to remain within the cell. IL-1 Beta secretion from human monocytes was significantly reduced by deletion of the 19 kDa gene (p < 0.02). Complementation by the wild type, but not the mutagenised gene reversed this phenotype. The effect of deletion and complementation on IL-12p40 and TNF secretion was less marked with no statistically significant differences between strains. Although deletion of the 19 kDa reduced apoptosis, an effect that could also only be reversed by complementation with the wild type gene, the results were variable between donors and did not achieve statistical significance.

**Conclusion:**

These results confirm in the context of the whole bacillus an important role for post-translational modification of the 19 kDa on both the cellular location and immune response to this protein.

## Background

The vast increase in knowledge that has accompanied the discovery of microbial pattern recognition receptors has focussed research into the microbial ligands that initiate these cellular responses [1,2] For example it is now known that bacterial LPS triggers responses via Toll like receptor (TLR) 4, and Flagellin via TLR5 [3,4]. It is also increasingly appreciated that receptors may co-operate to recognise specific ligands [5]. Thus triacylated lipopeptide is recognised by a heterodimer of TLR2 and 1, with diacylated lipopeptide being recognised by the TLR2/6 heterodimer [2]. Many types of pathogens produce lipoproteins and are thus in part recognised by TLR2 [6-8].

*Mycobacterium tuberculosis *has over 100 probable or known lipoproteins, many of which are concentrated in the cell wall [9]. Whilst a role has been assigned to some of these proteins (e.g. Phosphate binding and transport for the *Pst*S1-3 group [10]), most have not been assigned a function. They are characterised by an acylated N-terminus, processing of which is mediated by the consecutive activity of prolipoprotein diacylglyceryl transferase (*Lgt*) and lipoprotein signal peptidase (*Lsp*A) [11]. Deletion of LspA reduces the virulence of *M. tuberculosis*. In addition many of the lipoproteins have been found to be targets of both the innate and acquired immune response. A prominent target of the innate response is the 19 kDa lipoprotein encoded by Rv3763. This molecule has been intensively researched because of its pleiotropic effects on the innate immune response that include induction of cytokine genes, bacterial killing, induction of apoptosis, and the downregulation of Interferon-γ (IFN-γ) induced MHC Class II expression [12-20]. More recently it has also been suggested that the 19 kDa protein acts an adhesin [21].

Many of the above studies of the 19 kDa were performed with purified or recombinant protein that may not fully reflect the role of the molecule in the context of natural infection. In particular expression in *E. coli *is unlikely to reproduce native patterns of post-translational modiufication. We have previously reported the effect of deletion and overexpression of the 19 kDa on the innate immune response [22]. We found that the deletion mutant (Δ19) was moderately impaired in its ability to multiply in human monocyte-derived macrophages (MDM). Surface expression of MHC class II molecules was reduced in phagocytes infected with MTB; this effect was not seen in cells infected with Δ19. Δ19 induced lower IL-1β secretion from monocytes and MDM. Overexpression of the 19 kDa increased IL-1β, IL-12p40 and TNF-α secretion irrespective of phagocyte maturity. These findings confirmed the 19 kDa protein to be an important mediator of the innate immune response in the context of the whole bacillus.

In addition to being acylated, the 19 kDa protein is glycosylated [23,24]. Earlier work in our laboratories established that poly threonine motifs towards the N-terminal of the molecule form a major glycosylation site [23,24]. The aim of this study was therefore to evaluate the innate immune response to Δ19 mutants that had been complemented with a single copy of mutagenised 19 kDa molecules lacking the motifs for acylation and *O*-glycosylation respectively.

## Methods

### Generation of recombinant strains of *M. tuberculosis*

The 19 kDa gene was deleted from *M. tuberculosis *(MTB) H37Rv to produce the Δ19 strain as previously described [22]. Complementation of the Δ19 strain by the native and modified (non-acylated NA, and non-*O*-glycosylated NOG) 19 kDa genes led to the strains Δ19::19, Δ19::19NA and Δ19::19NOG. For complementation, the native sequence (including the entire intergenic region and part of upstream Rv3762 ORF) was amplified by PCR from H37Rv DNA. The site-directed mutagenised genes were amplified from previous episomal constructs [24,25] engineered to come under the control of the endogenous 19 kDa promoter. Complementation was performed using the integrating vector pKINTA, based on the L5 phage integration system [26], which reintroduces a single copy of the 19 kDa gene into the chromsome under the control of its own promoter at the *att*B site [27]. PCR was used to confirm deletion and insertion as previously described [22] and sequencing of the pKINTA insert confirmed nucleotide differences that would result in substitution of the N-terminal cysteine residue of the mature wild-type protein with alanine in Δ19::19NA; and substitution of two threonine clusters (5 amino acids in total) by valine residues in Δ19::19NOG. For Western blotting supernatants and sonicated preparations of wild-type *M. tuberculosis *H37Rv and the deleted and complemented strains were fractionated by SDS-PAGE and expression of the 19 kDa antigen compared by Western blot analysis using a polyclonal anti-19 kDa serum.

### Isolation and culture of monocytes

Buffy coats from healthy donors were obtained from the National Blood Transfusion Service (Colindale, London, UK). Following dilution in RPMI (1/3 vol/vol), peripheral blood mononuclear cells (PBMC) were separated by centrifugation over Ficoll-Paque Plus (Pharmacia, Uppsala, Sweden). Cells were washed in RPMI and counted. Cells were suspended at 1.2 × 10^7^/ml in RPMI/10% FCS medium and aliquots of 25 mls were added to 150 cm^2 ^tissue culture flasks. Flasks were placed flat in a 5% CO_2 _incubator and monocytes allowed to adhere for 2 h at 37°C. Non-adherent cells were removed by washing 3 times with 10 mls of pre-warmed RPMI. Finally, 10 mls of ice-cold PBS was added and the flasks were incubated at 4°C for 20 mins. Using a scraper, monocytes were gently dislodged from the bottom of the flasks and pooled in a 50 ml Falcon tube to count. Cells were plated in RPMI containing 10% serum at 10^6^/well in a 24-well tissue culture plate, and cultured overnight before infection.

### Infection of cells

Bacilli used to infect cells were grown in Middlebrook 7H9 broth supplemented with ADC to mid-log phase (OD 0.4–0.8) then frozen in aliquots in 15% glycerol. The CFU content of aliquots was determined by serial dilution and plating on Middlebrook 7H11 agar supplemented with OADC. Monocytes were infected at a multiplicity of infection of 1:1 without removing non-phagocytosed bacteria. Culture duration was 72 hrs., at which time supernatants were aspirated, 0.22 μm filtered, and stored at -80°C pending analysis by ELISA.

### ELISA

Cytokine ELISA was performed using the DuoSet ELISA Development Systems (R&D Systems, Minneapolis, MN) following the manufacturer's recommendations. The sensitivity of the assays was 15 pg/ml for IL-12p40, 10 pg/ml for IL-1β and 50 pg/ml for TNF-α. Histone associated DNA fragments, released into tissue culture supernatant and interpreted as evidence of apoptotic cell death, were assayed by the cell death detection ELISA (Roche Applied Science, Lewes, Sussex, UK) according to the manufacturer's instructions.

### Sequence analysis

Homologues of the *M. tuberculosis *19 kDa gene LpqH were identified by Blast searches of sequenced genomes [28]. Alignment of protein sequences was performed using Clustal W and results are displayed as a sequence pile-up and as a neighbour-joining tree. Strains and genome accession numbers: *M. tuberculosis *H37Rv, AL123456.2; *M. smegmatis *MC^2^155, CP000480.1; *M. ulcerans *Agy99, CP000325.1; *M. marinum *M, CP000854.1; *M. leprae *TN, AL450380.1; *M. avium subsp. paratuberculosis *K-10, AE016958.1; *M. abscessus*, CU458896.1; *Nocardia farcinica *IFM 10152, AP006618.1; *Rhodococcus *sp. RHA1, CP000431.1.

### Statistical methods

Paired and unpaired parametric variables were compared by student's t-test. Paired and unpaired non-parametric variables were compared by Wilcoxon signed rank or Mann Whitney U test respectively. Significance was inferred for p values ≤ 0.05.

## Results

### Bioinformatic analysis of 19 kDa genes in various mycobacteria

**Figure 1:**
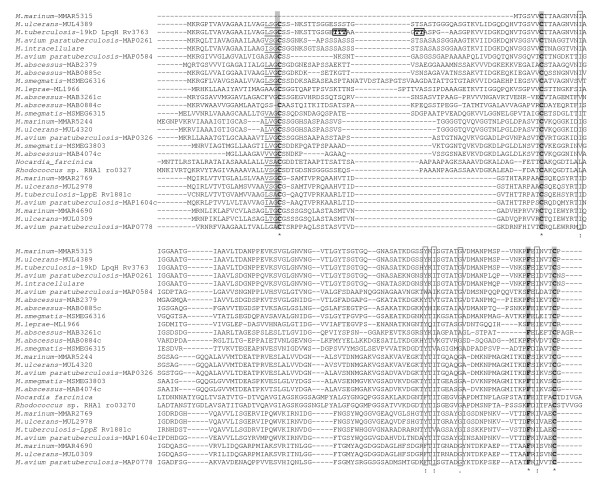
**Sequence alignment of 27 open reading frames belonging to the 19 kDa family**. Highly conserved cysteine, and phenylalanine residues are highlighted. "*" indicates fully conserved positions; ":" indicates strong conservation; "." Indicates weaker conservation. The 0-glycosylated threonine residues in the *M. tuberculosis *LpqH are boxed. Fully compliant Lipobox acylation motifs are underlined.

**Figure 2:**
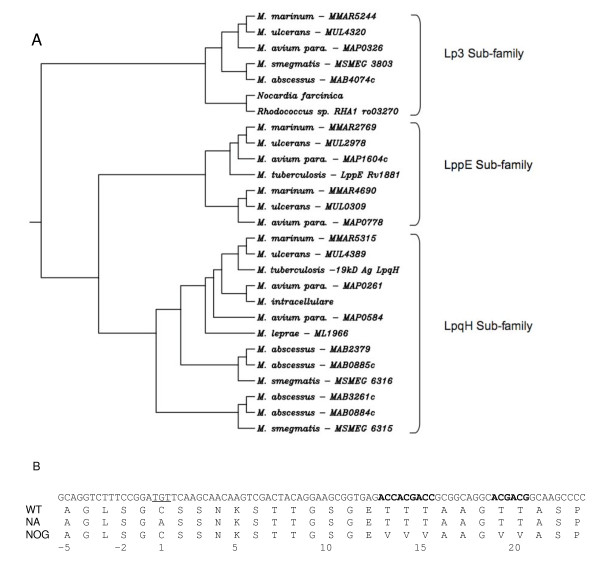
**A. Neighbour-joining tree of 19 kDa homologs**. Family members are found in both slow-growing and fast-growing mycobacteria and in the closely related genera, *Nocardia *and *Rhodococcus*. The predicted 19 kDa proteins fall into three sub-families: LpqH, LppE and Lp3. B. Nucleotide sequence of the N-terminal coding sequence of the 19 kDa gene indicating the sequences that were modified in the Δ19 strains complemented by the non-acylated or non-*O*-glycosylated 19 kDa molecule. The disruption to sequence encoding the N-Acyl diglyceride motif is indicated by underlined text and the disruption of the 2 threonine clusters shown in **bold**. The protein sequence of the wild type and each variant is also shown. Amino acid numbering is based upon the mature protein after cleavage of the signal peptide.

The 19 kDa or LpqH lipoprotein of *M. tuberculosis *belongs to a family of conserved proteins that is ubiquitous through the mycobacteria and is also found in the closely related *Nocardia farcinica *and *Rhodococcus *but not in other high GC gram positive bacteria such as *Streptomyces *and *Corynebacteria*. In addition to the *lpqH *gene, *M. tuberculosis *possesses a paralogous gene encoding the lipoprotein LppE. Other mycobacteria have varying numbers of 19 kDa gene homologs with the fast-growing *M. abscessus *possessing 6 paralogous genes. Figure 1 shows an alignment of twenty seven 19 kDa family proteins identified from genome sequencing projects. Displayed as a neighbour-joining tree, it is apparent that the 19 kDa proteins fall into three general sub-families: LpqH-like proteins, LppE-like proteins and a third subfamily that we term Lp3 (Figure 2A). All except one protein (the *M. marinum *MMAR5315 protein is truncated) contain a predicted secretion signal sequence with the N-terminus of mature proteins containing a cysteine residue. Twenty-one out of twenty-six predicted full-length 19 kDa proteins including the *M. tuberculosis *LpqH and LppE proteins, comply with the lipobox consensus acylation motif [29]. This is consistent with the approximately 75% predictive value of the lipobox based on experimental evidence of known prokaryote lipoproteins. Cysteine residues at positions 67 and 158 (relative to the *M. tuberculosis *sequence) and phenylalanine at position 152 are conserved throughout the family. Strongly and weakly conserved groups of amino acids are also highlighted in Figure 2B. *O*-glycosylation does not occur at a particular motif of amino acids but occurs at specific residues, generally threonine and serine. The *M. tuberculosis *LpqH 19 kDa protein is glycosylated at a triplet and a pair of threonines at positions 14–16 (relative to the start of the mature protein) and 19–20 [24]. Threonine pairs are also found in several other 19 kDa family proteins including, for example, the predicted protein from *N. farcinica *which has two pairs of threonine residues at positions 11–12 and 15–16. In addition, many of the 19 kDa homologs have N-terminal regions of the mature protein that are rich in serine residues which may be indicative of glycosylation. Taken together, it seems likely that N-terminal glycosylation and acylation are general features of the 19 kDa protein family. The broad distribution of this family across mycobacteria and closely related genera suggests that these lipoproteins fulfil some conserved physiological function which at present remains largely unknown. To screen for a possible role for the 19 kDa lipoprotein in mycobacterial physiology, we therefore generated a deletion mutant lacking the 19 kDa molecule and complemented this mutant with the wild type and site-mutagenised copies of the 19 kDa molecule.

### Generation and characterization of recombinant *M. tuberculosis *strains

**Figure 3:**
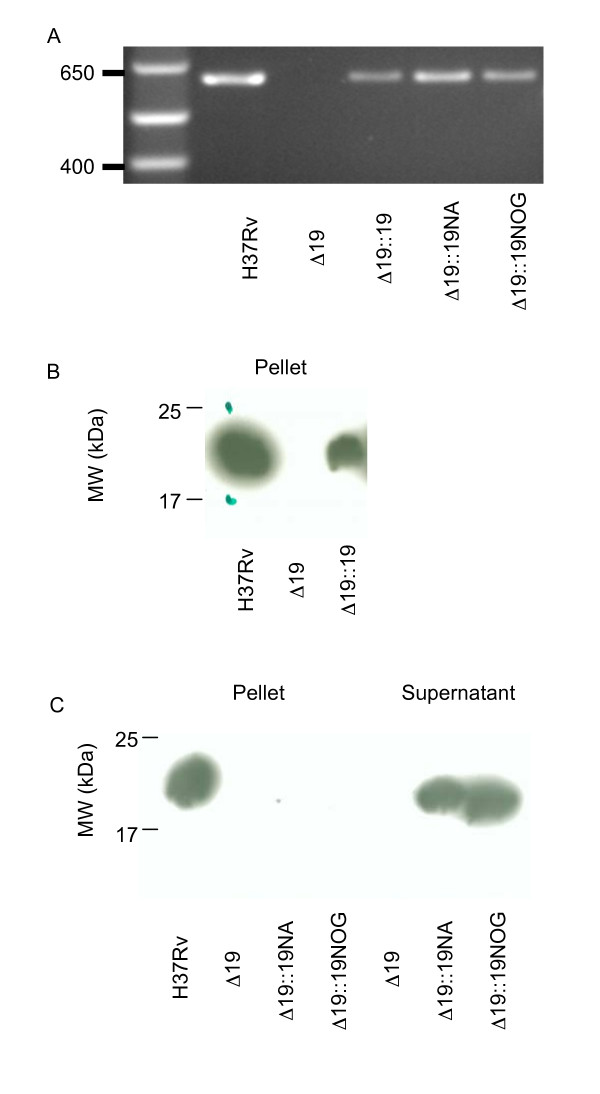
**Characterization of mutant *M. tuberculosis *strains**. A. PCR analysis showed Rv3763 to be absent from Δ19 and that this sequence had been successfully reintroduced into strains Δ19::19,, Δ19::19NA, and Δ19::19NOG. B. Western Blotting of cellular pellet indicating that the 19 kDa is not produced in Δ19 (lane 2). Expression of native protein of the same MW is restored close to normal levels by reintroduction of the 19 kDa gene in strain Δ19::19. C. Analysis of pellet and culture supernatant of complemented mutant strains. 19 kDa protein was only detected in the supernatant of cultures of the non-acylated (NA) and non-*O-*glycosylated complemented strains and was of slightly lower MW than the native 19 kDa.

**Figure 4:**
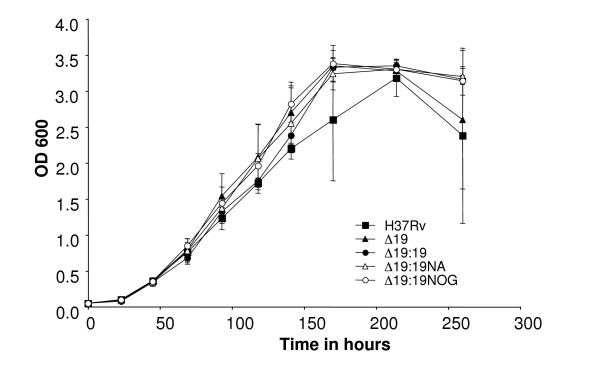
**Growth of strains in Middlebrook 7H9 broth**. Duplicate log phase cultures of each strain were normalised to an O.D. of 0.05 and cultured with shaking with the O.D. repeated at intervals. No difference in the maximum rate of growth of the strains was observed.

PCR analysis showed Rv3763 to be absent from Δ19 and that this sequence had been successfully reintroduced into strains Δ19::19,, Δ19::19NA, and Δ19::19NOG (Figure 3A). Western Blotting of cellular pellet indicated that the 19 kDa was not produced in Δ19 (Figure 3B, lane 2). Expression of native protein of the same MW was restored close to normal levels by reintroduction of the 19 kDa gene in strain Δ19::19 (Figure 3B, lane 3). 19 kDa protein was only detected in the supernatant of cultures of the non-acylated (NA) and non-*O-*glycosylated complemented strains and was of slightly lower MW than the native 19 kDa. In Middlebrook 7H9 broth the growth rate of the Δ19, Δ19::19, Δ19::19NA, and Δ19::19NOG strains was identical (Figure 4).

### Cytokine secretion

**Figure 5:**
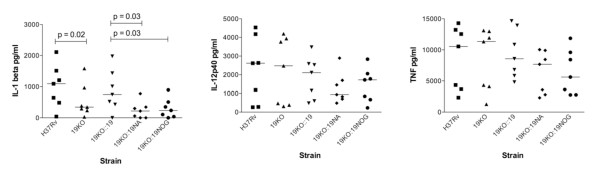
**Secretion of IL-1β, IL-12p40 and TNF in response to strains of *M. tuberculosis***. Monocytes from 7 donors were infected with strains and co-cultured for 72 hours. The median secretion of IL-1β was significantly reduced by deletion of the 19 kDa gene (p = 0.02). Introduction of the native 19 kDa gene as Δ19::19 restored secretion to wild type levels but the response to Δ19::19NA and Δ19::19NOG remained significantly less when compared to Δ19::19 (p = 0.031 in both cases). No differences existed between strains in their ability to induce the secretion of IL-12p40 or TNF.

Human monocytes were infected with equal numbers of bacilli (moi 1:1) and co-cultured for 72 hours. During this period, the median secretion of IL-1β was significantly reduced by deletion of the 19 kDa gene (Figure 5A, p = 0.02). Introduction of the native 19 kDa gene as Δ19::19 restored secretion to wild type levels but the response to Δ19::19NA and Δ19::19NOG remained significantly less when compared to Δ19::19 (p = 0.031 in both cases). There was no difference between H37Rv, Δ19 and Δ19::19 in their ability to elicit IL-12p40 or TNF from monocytes (Figure 5B and 5C). Although the response to both the Δ19::19NA and Δ19::19NOG strains tended to be lower, these differences were also not significantly different from H37Rv.

### Induction of apoptosis

**Figure 6:**
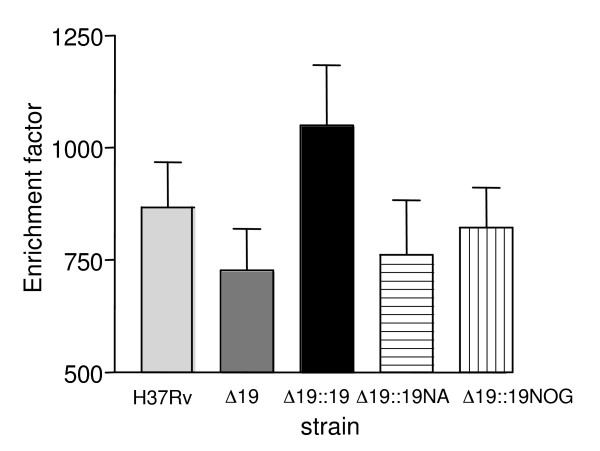
**Induction of apoptosis by strains of *M. tuberculosis***. Monocytes from 6 donors were infected with strains and co-cultured for 72 hours. Apoptosis was determined by ELISA for nucleosomal fractions in culture supernatants. Results for each subject were normalised to unstimulated cells to generate an enrichment factor. The mean + SD of this enrichment factor is shown. Although the Δ19 strain tended to induce less apoptosis than H37Rv and Δ19::19 none of the differences were statistically significant.

Culture supernatants from 6 donors were also assayed for the presence of Histone associated DNA fragments, a marker of apoptosis. Results for each subject were normalised to unstimulated cells to generate an enrichment factor. The Δ19 and Δ19::19NA and Δ19::19NOG were associated with lower levels than H37Rv or the Δ19::19 strain. However responses varied considerably between donors and none of these trends attained statistical significance (Figure 6).

## Discussion

We have investigated deletion of the 19 kDa lipoprotein (Rv3763) from *M. tuberculosis *and chromosomal complementation of the deletion mutant by the wild type gene and site directed mutagenised variants lacking motifs for acylation and *O*-glycosylation. We have determined that both acylation and *O*-glycosylation are necessary for the protein to remain within the cell. Consistent with our previous findings, the 19 kDa is an important stimulus for the production of pro-inflammatory IL-1β, an effect that is dependent on acylation and *O*-glycosylation. The effect of deletion and complementation on IL-12p40 and TNF secretion was less marked with no statistically significant differences between strains. Although deletion of the 19 kDa reduced apoptosis, an effect that could also only be reversed by complementation with the wild type gene, the results were variable between donors and did not attain statistical significance.

An interesting finding was that 19 kDa protein was only detected in the supernatant of cultures of the non-acylated (NA) and non-*O-*glycosylated complemented strains, whereas the Δ19::19 strain expressed the molecule in both pellet and supernatant. This suggests that in order to be retained within the cell wall both acylation and glycosylation are necessary for anchoring within the cell wall. Whether this relates to a specific physicochemical interaction or merely reflects the recognised hydrophobicity of the mycobacterial cell membrane remains to be determined. Sartain and Belisle have recently shown that *o-*glycosylation affects the positioning in the cell wall but not the enzymatic activity of the superoxide dismuase *sod*C [30].

In a previous study overexpression of the 19 kDa in *M. smegmatis *reduced its capacity to induce the secretion of IL-12p40 and TNF[18]. This effect was dependent on acylation and glycosylation, as tranformation of, *M. smegmatis *with NA and NOG variants of the 19 kDa did not reduce the secretion of these cytokines. By contrast overexpression of the native 19 kDa molecule in Δ19 strain of virulent *M. tuberculosis *had precisely the opposite effect, with the production of IL-12p40 and TNF increased irrespective of phagocyte maturity [22]. In this study we reintroduced the 19 kDa gene as a single copy into the chromosome of H37Rv under the control of its own promoter. We precisely reproduced our previous findings with respect to the effect of deletion of the 19 kDa on the cytokine response of monocytes. We have shown that the 19 kDa mediated induction of IL-1β is dependent on acylation and glycosylation. Taken together these and other studies suggest a consistent effect of acylation and *O*-glycosylation on the cytokine response to the 19 kDa, but that the genetic background and level of expression are also important. Further evidence in favour of this hypothesis is our recent finding that a naturally occuring *M. tuberculosis *strain that lacks the 19 kDa gene does not have the same *in vitro *phenotype as the engineered knock out on the Rv background (data not shown). This potentially important finding requires further investigation as much of our knowledge about gene function in *M. tuberculosis *is inferred from studies of isogenic mutants on the H37Rv background.

Considerable evidence now points to the protective role of macrophage apoptosis in tuberculosis. Apoptosis may prevent the release of intracellular components and the spread of mycobacterial infection by sequestering the pathogens within apoptotic bodies [14,31,32]. In addition, uptake of apoptotic debris by competent phagocytes allows efficient cross-presentation of *M. tuberculosis *antigens [33]. Thus, the avoidance of apoptosis may be considered a virulence mechanism and a recent study has in fact reported a inverse relationship between the intracellular growth rate and the ability of strains to induce apoptosis [34]. Two previous studies have implicated the 19 kDa as pro-apoptotic [14,17] and our results, although variable between donors tend to support this conclusion. However the dependence or otherwise on post-translation modification requires additional work as the findings of Lopez *et al*. suggested that this effect was acylation independent, whereas the trend in our study suggest acylation is necessary (Figure 6).

## Conclusion

In conclusion we have presented further evidence of the role of the 19 kDa as a key modulator of the human innate immune response. There is considerable evidence that the protein downregulates IFN-γ induced macrophage activation, an effect that will tend to favour bacillary survival during the development of an acquired immune response. On the other hand the molecule will tend to give away the presence of bacilli to the innate system early in infection, perhaps teleologically explaining why it is not upregulated early after infection [22]. In addition, this work provides further evidence of the utility of defined mutants to delineate key determinants of the innate immune response in the context of whole bacilli.

## Authors' contributions

KAW, RJW, GRS and DBY designed the research. RJW, GRS and SMS derived the recombinant strains using constructs designed and prepared by ON and J-LH. KAW and SMN performed and interpreted the immunological studies. GRS performed the bioinformatic analysis. All authors contributed to analysis and to writing the manuscript.
